# Telesurgery – an efficient interdisciplinary approach used to improve the health care system

**Published:** 2014

**Authors:** C Cazac, G Radu

**Affiliations:** * “Carol Davila” University of Medicine and Pharmacy, Bucharest, Romania

**Keywords:** Telesurgery, Asynchronous transfer mode, virtual private network

## Abstract

At the time of the writing of this article, there are three operational telemedicine control centers in Bucharest, Targul-Mures, and Iasi; however, the current telemedicine infrastructure has limited geographic coverage and is exclusively used in the field of emergency medicine with only few promising beginnings in the domain of family medicine. Nevertheless, many areas of Romania are still lacking qualified medical personnel that can perform vital surgeries thus requiring patients to travel long distances to obtain the health care services they require.

In order to improve the current healthcare infrastructure and eliminate the difficulties associated with a lack of qualified medical personnel in rural areas of the country, this article suggests the implementation of telesurgery as a practical solution. This article will hope to analyze the applicability of telesurgery by looking at the benefits and costs of creating a national telesurgery infrastructure, by predicting possible obstacles in creating such a system and by suggesting ways in which these obstacles can be avoided. The writing of this article was possible thanks to interviews, articles, and data obtained from surgeons and medical personnel that practice in Romania, the Republic of Moldova, Canada, and the United States of America. A vast majority of technical details has been furnished by the producers of robotic surgery platforms such as Intuitive Surgical®.

## Introduction

The first robotic surgery was conducted in 1985 with the help of a robotic arm called Puma 560 that was used for non-laparoscopic neurosurgical biopsies [**1**-**3**,**8**]. Less than 20 years later, in 2002, the first robot-assisted transatlantic telesurgery was performed by using Asynchronous Transfer Mode (ATM) with a constant rate of data transfer of 54 bytes and land networks that spanned a distance of over 14,000 km between Manhattan, New York, and Strasbourg, France [**4**]. The abovementioned surgery was a cholecystectomy with a total operation duration of 1 hour, 54 minutes and an additional 16 minutes used to set up the surgical apparatus and for trocar placement [**4**]. The procedure was successfully performed on a 68 year old patient with a medical history of cholelitiasis [**4**]. During the operation there were no significant complications, the patient recovered well from anesthesia and there were no post-operative incidents [**4**]. This revolutionary procedure sparked further interested in telesurgery and led to the creation of numerous other studies. These studies have tried to reduce the barriers between science and science-fiction by introducing the concept of international telesurgery to the domain of medical science. Even more so, Marescaux’s results have shown that telesurgery can shatter any geographical barriers between physician and patient; therefore, if distances of over 14,000 km can be conquered with a infrastructure supporting ATM technology and a robotic surgery apparatus then the implementation of such a system in Romania, where the geographical distance between the capital Bucharest and any other point in the country is less than 600 km, is possible. 

## Discussion

Patients from rural settings travel at least 1.5 hours to receive surgical procedures that they require, but the majority of these patients, for various reasons, cannot travel long distances, which in consequence leads to the fact that patients in areas where there is a lack of medical personnel do not receive the medical care that they need. On the other side, the doctors that decide to travel to areas lacking qualified medical personnel to provide medical services expose themselves to potential costs as well as to a heightened degree of risk associated with travel. Although numerous agencies have tried to reduce the geographical barriers between doctors and patients by creating national programs like IPSR, [**10**] the short-term and long-term costs of the absence of an efficient medical infrastructure are colossal. For example, the estimated cost of a lack of a specialized network used for telesurgery has costed the Romanian state over 8,050,000 euros in damages for the year 2014 when a team of surgeons, who flew to perform a surgical liver extraction from a donor, crashed in the Apuseni Mountains on January 20, 2014 [**6**]. In the presence of a national telesurgical program, the necessity to expose doctors to a heightened risk can be avoided.

Next, since the ATM technology used to perform the first transatlantic surgery is not very cost-efficient to implement in present times because the annual cost for 1 year of service ranges from 100,000 to 200,000 U.S. dollars [**4**,**5**], and is generally not available in rural areas, it is a poor candidate for the implementation in a nationwide program. Nevertheless, it is important to mention that it is not necessary to use ATM technology to create an effective network for telesurgery for distances lower than 1000 km since M. Anvari has proved, through the creation in 2003 of a working telesurgery platform that links a rural and a urban hospital in Canada, that commercially available networks such as Virtual Private Networks (VPM) can be successfully used for the purpose of telesurgery [**11**]. The hospitals in Canada that are currently practicing telesurgery through VPM are the St. Joseph Hospital in Hamilton, Ontario, Canada, and North Bay General Hospital situated in the city of North Bay, Ontario, Canada located at a distance of 400 km from one another [**11**].

In the interval between 2003-2005, the telesurgical system mentioned above has been successfully used to conduct 21 robot-assisted surgeries at a distance of which: 13 fundoplications, 3 sigmoid resections, 2 right hemicolectomies, 1 anterior resection and 2 inguinal hernia repairs [**11**]. All of those procedures have commonly used a IP-VPN system with 15Mbps of bandwidth without having encountered any significant surgical complications or an increase in post-operative stay [**11**]. As a result of this improvement in the Canadian medical infrastructure, telesurgery is now routinely used [**11**].

## Is there a need for telesurgery in Romania? 

In the year 2010, 580 surgeries have been conducted using robotic platforms of which 400 were conducted at the Fundeni Institute in Bucharest, 100 at the Floreasca Hospital in Bucharest, and 80 in Cluj [**14**,**15**].

Although the number of robotic surgeries has almost doubled to 150 in 2011 using the DaVinci platform in Cluj, Dr. Nicolae Constantea estimates that there is an approximate need of 300 surgeries using this platform per year [**12**]. Also, the patients from Cluj are on a waitlist for approximately 3 weeks for surgical interventions using the DaVinci platform [**12**]. So, there is an immediate need to create a national telesurgery infrastructure in which patients can benefit from the treatments that they need. 

**Fig. 1:**
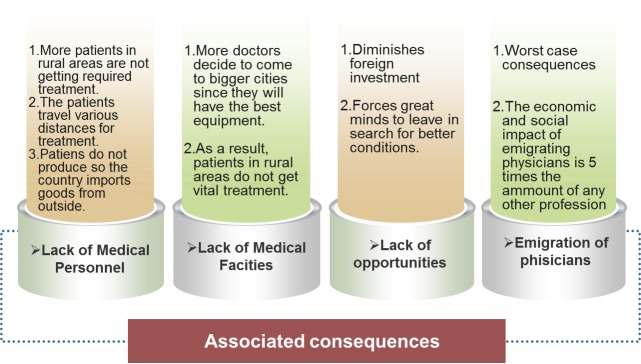
Examples of long-term consequences of the lack of a telesurgery system

Additionally, Romania is facing multiple challenges in the current health care system among which are: (1) lack of qualified medical personnel (2) lack of medical equipment (3) lack of medical consumables (4) lack of research opportunities (5) lack of jobs and (6) emigration of physicians. A hypothetical example of the consequences of these deficits can be seen in **Fig. 1**. A part of these challenges, or all of them, can be remedied by bettering the current medical infrastructure. Creating a telesurgical system can also open doors to international surgical collaboration thus producing an increase in the number of physicians practicing in Romania.

Furthermore, in order to create an effective telesurgical infrastructure, it is necessary to invest in the acquisition of medical equipment thereby solving the problem of the lack of medical equipment. The problem of the lack of medical consumables can be partially eliminated through the use of interchangeable telesurgical instruments. In addition, a national system of telesurgery can open a new horizon of research opportunities in this interdisciplinary domain of medical informatics and applied medicine in which Romania can easily become a pioneer. It is also implied that the introduction of this vast array of improvements has the potential to create new jobs and reduce the percentage of emigration of medical doctors.

**Financial costs of implementing telesurgery**

**Table 1:** Alternative costs of implementation of telesurgery in Romania. The price of the used but functional surgical robot DaVinci IS-1200 produced in 2005, including the control station, is included in the minimum section [**7**]. The price of a new and functional surgical robot DaVinci Si produced in 2012 is included in the maximum section [**7**]. The prices are actual from www.medwow.com as of 7/11/2014.

Initial costs		Operational Costs	
The cost of ONE robotic surgery platform	Minimum (used) € 16, 032.05 [**9**]	The cost for ONE surgery	
	Maximum (new) € 803,379.84 [**9**]	• Consumables	€3,564.15 [**12**]
		• Mandatory maintenance	€ 735.46 [**12**]
		• Total Costs per procedure	€ 4299.61 [**12**]
Additional costs of network	€ 2,500	Cost per 150 surgeries	€ 644,850
Cost of VPN network	€ 1000	Cost of ATM technology for 1 year	€ 100,000-200,000 [**4**]
		Cost of VPN technology for 1 year	Variable €100.00 – 200.00
Total	Minimum € 39,032.05	Total	Minimum € 645,000
	Maximum € 315, 522.9		Maximum € 744,850

According to **Table 1**, the total costs for creating and maintaining a functional telesurgery platform for one year is estimated to be **€ 903,111** while the cost of a lack of telesurgical infrastructure rises well above € 8,050,000 (8 times more than the cost of telesurgery per 1 year).

**The benefits of robotic surgery to the surgeon [**13**].**

Patients, as well as doctors state that they prefer robotic surgical procedures to traditional surgical methods. 

Among the benefits of robotic surgery there are: 

1) Reduction to elimination of surgical physiological tremor

2) Increased maneuverability around small blood vessels

3) Reduced percentage of damaged tissue after surgical procedure

4) Faster post-operative recovery

5) Increased surgical precision

6) Increased number in patients with the potential for treating international patients without leaving the hospital

7) Better allocation of financial and time resources

8) Reduced risk of travelling to remote areas of the country

9) Increased surgical collaboration between surgeons

10) Increased national level of health 

11) Creation of new jobs and increasing the possibilities of research

12) Creation of an opportunity for investment

13) Acquisition of new knowledge and new surgical skills.

**The benefits of robotic surgery and telesurgery for the patient [**13**].**

1) Patients benefit from national coverage.

2) Patients do not need to travel long distances to get medical care.

3) Patients can benefit from the services of a better-qualified surgeon whom in normal conditions they could not have contacted due to geographical distances.

4) Faster post-operative recovery with the corresponding reduction in costs and increase in the efficiency of the hospital

**Fig. 2:**
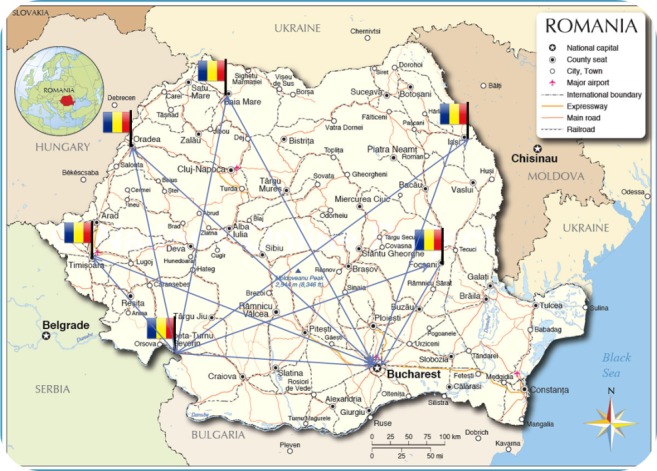
Telesurgical infrastructure in Romania. In the above image, every flag represents a telemedicine control station that can be equipped with the necessary equipment to conduct telesurgery. Every control station is placed strategically to allow a maximal coverage of Romania.

An example of a telesurgery infrastructure is presented in **Fig. 2**].

**Ethical Considerations**

Any form of surgical intervention requires both the protection of the patient as well as the protection of the surgeon from cases of medical malpractice. In the case of Canadian telesurgery between St. Joseph Hospital and North Bay Hospital, any form of telesurgery is performed only after obtaining the approval of the Ethics Board of both hospitals [**11**].]. A special information and consent sheet was developed by the Ethics Board for the patients who decide to undergo telesurgery [**11**]. Also, the surgeons are insured completely by the Canadian Medical Protection Association.

**Fig. 3 A,B:**
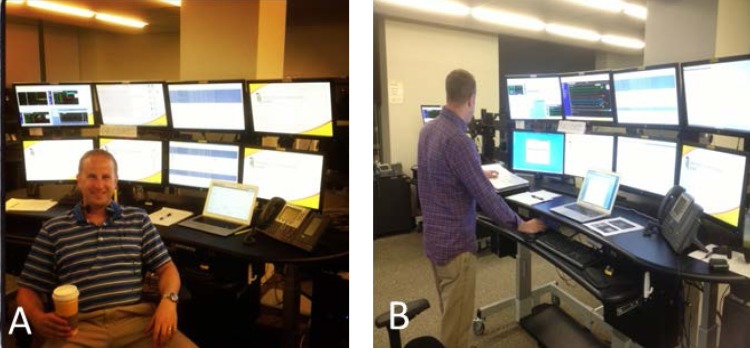
Telemedicine system used by Dr. David A. Vitberg, MD, in the Critical Care Services, GBMC, Baltimore, Maryland. Clinical Assistant Professor, Department of Emergency Medicine, University of Maryland School of Medicine, Associate Medical Director, Baltimore County Fire Department. Images reproduced with the permission of Dr. David A. Vitberg.

**International Collaboration**

After an interview with Dr. David A. Vitberg, Director Critical Care Services, Baltimore, Maryland (**Fig. 3**), Dr. Anatol Cazac, Surgeon, Chisinau, Republic of Moldova, and Dr. Iura Turcanu, Surgeon, Bucharest, Romania, it has been noted that there is a heightened desire for collaboration between physicians to implement a working international telemedicine platform. This observation shows that telesurgery is generally desired and accepted as a solution by numerous physicians.

## Conclusions

Telesurgery is the next logical step toward improving the medical system in Romania. The concept of telesurgery is easier to implement in Romania because there already exists a working telemedicine infrastructure used in emergency medicine. The benefits of telesurgery to Romania far outweigh the costs.
